# Microbiota and probiotics: chances and challenges – a symposium report

**DOI:** 10.1017/gmb.2023.4

**Published:** 2023-03-27

**Authors:** Carrie Helen Stevenson Ruxton, Chiyuki Kajita, Paola Rocca, Bruno Pot

**Affiliations:** 1 Nutrition Communications, Cupar, UK; 2 Yakult Europe BV, Almere, Netherlands; 3 Yakult Italia Srl, Milan, Italy

**Keywords:** Parkinson’s disease, probiotics, infancy, ageing, fermented foods, irritable bowel syndrome

## Abstract

The 10th International Yakult Symposium was held in Milan, Italy, on 13–14 October 2022. Two keynote lectures covered the crewed journey to space and its implications for the human microbiome, and how current regulatory systems can be adapted and updated to ensure the safety of microorganisms used as probiotics or food processing ingredients. The remaining lectures were split into sections entitled “Chances” and “Challenges.” The “Chances” section explored opportunities for the science of probiotics and fermented foods to contribute to diverse areas of health such as irritable bowel syndrome, major depression, Parkinson’s disease, immune dysfunction, infant colic, intensive care, respiratory infections, and promoting healthy longevity. The “Challenges” section included selecting appropriate clinical trial participants and methodologies to minimise heterogeneity in responses, how to view probiotics in the context of One Health, adapting regulatory frameworks, and understanding how substances of bacterial origin can cross the blood–brain barrier. The symposium provided evidence from cutting-edge research that gut eubiosis is vital for human health and, like space, the microbiota deserves further exploration of its vast potential.

## Introduction

Decades of research have revealed the remarkable extent to which the gut microbiota (GM) influences and interacts with many areas of the body beyond the large intestine. Slowly, a picture has emerged of the potential role of the GM in helping to modulate gut health, immune function, mineral absorption, metabolic balance, appetite, brain health, and ageing.

This creates opportunities for the use of dietary or medical interventions which may impact the GM by promoting particular microbiological species, excluding others, or broadening microbiological diversity. It also poses challenges to understanding mechanisms, ideal intakes, appropriate health markers, and characteristics of responders, as well as how best to regulate products.

These were the topics considered by the 10th International Yakult Symposium held in Milan, Italy, on 13–14 October 2022. This report summarises the presentations given by a panel of international experts and invites reflection on the chances and challenges presented by the study of the GM and probiotics.

## The crewed journey to space and its implications for the human microbiome

Space travel is a unique environment in which to study the human microbiome. Prof. Christine Moissl-Eichinger from the Medical University of Graz, Austria, outlined why a good understanding of the GM is essential for ensuring the success of crewed space missions, mainly as 8 per cent of astronauts report gastrointestinal issues and access to medical interventions in space is limited.

### Simulation experiments

Space training in closed systems provides opportunities to study changes in the GM and those microorganisms present in the environment (Kuehnast et al., 2022). One example is the Mars 500 experiment which saw six crew members spend 520 days in a terrestrial-based simulator to mimic a journey to Mars (Schwendner et al., 2017). During this time, samples at different time points were taken from the surfaces and air of the module, revealing that microbial communities followed the functions of humans and could also be altered by human activity (eg. changing to a different cleaning product). This experiment also tracked the GM of the six crew members. Remarkably, given the constrained environment and similar diet, each person had their own signature GM which fluctuated over time but remained distinct from the GM of other crew members. Individual phyla, such as Pseudomonadota (formerly the Proteobacteria), Bacteroidota (formerly the Bacteriodetes), or Verrucomicrobiota (formerly Verrucomicrobia; Oren and Garrity, 2021) found in one person’s GM could be completely missing in the GM of others. In three subjects, major fluctuations in microbial configurations occurred after 340 days (range 330–360 days) in the module, which could be related to stress, or the tasks being performed. These fluctuations were characterised by the depletion of *Faecalibacterium prausnitzii, Ruminococcus bromii, Blautia luti, Anaerostipes hadrus,* and *Roseburia faecis.*

Another Mars simulation model is the Hawai’i Space Exploration Analog and Simulation (HI-SEAS) mission (Mahnert et al., 2021). This involved a team of astronauts spending 4–12 months in a 111 m^2^ module, during which time samples were taken from different areas of the module and the crew’s skin and faeces. Some interesting patterns emerged. Firstly, the microbial diversity reflected the function of the living area (eg. the toilet and kitchen). There was a crossover in the human microbiota when interactions occurred, such as a higher number of pathogens on the skin of the crew on toilet cleaning duty. Secondly, while each person had their microbiota signature, there were evident crossovers of species between those astronauts who had the most interactions with other crew members. Thirdly, while the microbiome of the built environment remained relatively stable over time, the skin microbiome of the crew increased in diversity as it incorporated species from the environment. This was particularly the case during an episode where a technical failure of the toilet facilities forced individual crew members to carry out additional cleaning duties, providing more chances for them to come into contact with faecal bacteria, which was then reflected in their skin microbiome.

### Experiments in space

Few studies have been conducted in space. In one of these, Mora et al. (2019) tested whether the unique conditions inside the International Space Station (ISS) altered the microorganisms found there. This is warranted since there is evidence that microgravity can influence the virulence of certain species (Rosenzweig et al., 2010), while technophilic microorganisms have been known to cause equipment to malfunction in space. The EXTREMOPHILES study involved sampling in several areas of the ISS over 3 months. The key learnings were:

- The diversity and composition of the ISS microbiome fluctuate in response to human activity reflecting the purpose of the different living areas but retaining a core group of stable species.
- The ISS microbiome is similar to indoor environments on Earth but has a greater prevalence of species that can make biofilms (for details, see Mora et al., 2019). This is probably due to adaptation to thrive on the metal surfaces inside the ISS.
- While the ISS microbiome was mostly human-associated, it was reassuring that no evidence was found of selection for enhanced pathogenicity or antimicrobial resistance (AMR) (Mora et al., 2019).

Further studies have found that space travel disrupts the normal GM, probably due to the influence of stress. During one space mission, astronauts’ skin, nose, and gut microbiomes changed markedly. The GM became more similar across crew members, primarily due to a drop in the abundance of several bacterial taxa mainly *Akkermansia*, *Ruminococcus*, *Pseudobutyrivibrio* and *Fusicatenibacter* (Voorhies et al., 2019). However, one longer-term study in twins (Garrett-Bakelman et al., 2019) found that the GM shifts back to the pre-flight pattern within 6 months of the astronaut returning to Earth.

To summarise, the microbiome of the built environment in space fluctuates around a set of core species but does not appear to present a particular risk to crew health in terms of pathogenicity, virulence, or antibiotic resistance. This is relevant as there are limited opportunities to treat microbial infections in space. While space travel disrupts humans’ normal skin and gut microbiome, this effect is reversible. Future space experiments will help find the answers to essential questions such as how to control microbial outbreaks in space, how to treat microbial disease in space, whether there is a need for novel probiotics/prebiotics, and how the microbiome of space environments and crew can be monitored long term at vast distances from Earth.

## Parkinson’s disease: evidence for the role of the gut

Initially viewed as a brain condition, there is growing evidence that the gut has a role in initiating Parkinson’s disease (PD), as discussed by Prof. Aletta Kraneveld from Utrecht University, The Netherlands.

PD affects 1 per cent of older adults and is an incurable condition characterised by progressive tremors, muscle rigidity, postural instability, and intestinal dysfunction. This conflation of gut and brain symptoms implies two origins for the accumulation of α-synuclein (Lewy bodies) in the brain leading to neuro-inflammation and neurodegeneration (Horsager et al., 2020): either a direct central nervous system phenotype, or an indirect intestinal phenotype where leaky gut and endotoxemia lead to mucosal inflammation, microbiome changes and, eventually, α-synuclein accumulation (Rietdijk et al., 2017; Scheperjans et al., 2018).

### Intestinal phenotype hypothesis

This is supported by more than 15 cohort studies which found correlations between neurological deterioration and gut dysbiosis characterised by reduced *Prevotella*, lower levels of faecal short-chain fatty acids (SCFAs), increased lipopolysaccharide- (LPS) producing bacteria, and increased pro-inflammatory Lactobacillaceae (Li et al., 2023). Murine models have built on this concept. Mice which genetically overexpress α-synuclein develop PD-related pathophysiology and motor dysfunction, but such changes do not occur if α-synuclein overexpression mice are bred germ-free. However, inoculating these germ-free mice with GM from PD patients induces the pathology to a greater extent than non-exposed α-synuclein overexpression mice, proving that gut bacteria are essential to initiating the disease (Sampson et al., 2016).

Other studies corroborate gut-related mechanisms. Colonising α-synuclein overexpression mice with *E. coli,* which produce curli fibres (pro-inflammatory proteins which mediate host cell adhesion and invasion), led to the further aggregation of α-synuclein in the gut and brain, and enhanced brain inflammation, gut problems, and motor dysfunction (Chapman et al., 2002). Another study (Matheoud et al., 2019) considered the role of the PINK1 gene, which is responsible for clearing mitochondria damaged during the progression of PD. Knocking out PINK1 would be expected to induce or exacerbate PD-like changes in animal models. However, this does not happen unless there is also an intestinal infection with LPS-producing bacteria.

A study of gut biopsies from PD patients revealed evidence of tight junction decline, leaky gut, and endotoxemia, and enhanced toll-like receptor (TLR) 4 expression, suggesting that PD is a TLR disease (Perez-Pardo et al., 2019). This hypothesis was tested using the pesticide, rotenone (an isoflavone molecule), which can initiate PD-like pathophysiology in animal models. Compared with wild-type mice which developed the expected pathogenic changes, oral exposure to rotenone for several weeks did not lead to gut dysbiosis or α-synuclein accumulation in TLR4 knock-out mice. In addition, the loss of dopamine-producing cells in the substantia nigra was less pronounced and there were fewer motor and cognitive problems. A similar disruption of the expected PD pathophysiology was seen following the use of TLR4 antagonists and TLR4 blocking antibodies, and when the vagus nerve was cut suggesting that this is the likely route by which α-synuclein spreads, prion-like, to the brain (Kim et al., 2019).

### Can GM modulation slow the progression of PD?

Faecal microbiota transplants (FMT) in murine models of PD reduce gut dysbiosis and neuroinflammation and result in fewer motor problems. Human trials are limited but demonstrate encouraging results for motor and non-motor symptoms in PD patients (Segal et al., 2021). Research on probiotics and synbiotics is more advanced and suggests that these are safe and effective, although further evidence is needed. *In vivo* studies report improved glucose metabolism, reduced inflammation, and neurodegeneration (Leta et al., 2021). In a review of eight clinical trials in PD patients given lactobacilli or bifidobacteria probiotics (Hong et al., 2022), constipation was significantly reduced, and modest anti-inflammatory effects were observed. A downside of using probiotics in PD is the potential for probiotic-drug interactions since bacterial decarboxylases may affect the bioavailability of l-dopamine, a Carbidopa component commonly used to manage PD neurological symptoms (van Kessel et al., 2019).

Murine PD models have been used to test the efficacy of diets containing precursors for neuronal membrane synthesis, such as long-chain omega-3 fatty acids, choline, uridine, vitamins, and minerals (Perez-Pardo et al., 2018b). Overall, the nutritional intervention was effective at partially alleviating the rotenone-induced neurological changes in mice. A further study tested an enhanced experimental diet containing the same nutrients as before plus prebiotic fibres but introduced it 28 days after rotenone exposure when adverse neurological changes had already occurred (Perez-Pardo et al., 2017). Compared with the control diet, the enhanced prebiotic-rich diet was more effective at normalising the mice’s rotenone-induced motor and non-motor abnormalities. These findings suggest that dietary treatments can help reverse neurological changes in mice and that diets that modulate the GM appear to deliver more benefits than those providing nutritional support (Perez-Pardo et al., 2018a).

In summary, there is growing evidence for a gut-first model of PD. However, further robust human studies in target populations are needed to understand the gut–brain mechanisms involved and identify opportunities for early intervention.

## Underlying mechanisms of depression and the modulating role of probiotics

Another condition potentially influenced by the gut–brain axis is major depressive disorder (MDD), which affects around 280 million people worldwide and is characterised by symptoms including depressed mood, anxiety, and insomnia. Dr Kazunori Matsuda, from the Yakult Central Institute, Japan, proposed underlying mechanisms related to the GM and the therapeutic potential of probiotics.

### Gut–brain axis

Bidirectional communication exists between the GM and the brain. The brain influences the gut via the autonomic nervous system, while the gut, including microbe-derived molecules, influences the brain via humoral and neuronal pathways (summarised in Suda and Matsuda, 2022). The idea that the GM could be linked to depression arose from studies where mice receiving an FMT from MDD patients displayed depression-like behaviour compared to control mice given an FMT from healthy people (Zheng et al., 2016), which indicates the role of dysbiosis in MDD development.

Further evidence came from a systematic review of 17 studies characterising the GM of MDD patients (Knudsen et al., 2021), which found reduced numbers of *Faecalibacterium*, a producer of butyrate, a SCFA linked to the maintenance of neurogenesis and anti-inflammatory effects. Other work reported that MDD patients have a lower abundance of bifidobacteria and lactobacilli than healthy controls (Aizawa et al., 2016). However, this is not a consistent pattern across studies, perhaps due to differences in subjects’ backgrounds.

### Mechanisms

What are the likely mechanisms if gut dysbiosis were influential in the pathology of depression? MDD is recognised as a multifactorial condition linked to abnormal stress response, reduced neurogenesis, and neuroinflammation, pathways where the GM may impact. Chronic stress is a risk factor for MDD onset, resulting in the hypothalamus–pituitary–adrenal (HPA) axis-mediated dysregulation of the stress response. The HPA axis is understood to be a key pathway of stress response through cortisol secretion. Normally, cortisol regulates its secretion via negative feedback through the HPA. However, in MDD patients, the feedback system is impaired, resulting in elevated blood cortisol. Brain exposure to high levels of cortisol induces chronic inflammation and reduced brain-derived neurotrophic factor (BDNF) protein – an important regulator of neuronal growth, survival, and plasticity.

Animal studies have found that the stress response is pronounced with a lack of GM when germ-free mice are exposed to physical restraint stress. However, when germ-free mice were inoculated with *Bifidobacterium infantis*, the exaggerated HPA stress response was reversed (Sudo et al., 2004). One signalling route from the gut to the brain is the vagus nerve, and some probiotic strains such as *Lacticaseibacillus paracasei* strain Shirota (LcS) have been shown to stimulate the activity of the gastric branch of the vagal afferent to suppress the stress-induced increase in blood corticosterone (Takada et al., 2016).

Reduced neurogenesis, another part of the pathophysiology of MDD, is believed to be caused by neuroinflammation and excessive stress, demonstrated by a smaller volume of certain brain regions in MDD patients (Treadway et al., 2015) and lower BDNF in cerebrospinal fluid (Mizui et al., 2019). This may have a GM link since germ-free mice have lower hippocampal levels of BDNF relative to specific pathogen-free mice (Sudo et al., 2004), and SCFAs can upregulate BDNF. Neuroinflammation, too, has a gut connection since the GM directly affects pro- and anti-inflammatory responses in the gut, and a leaky gut has been implicated in the pathogenesis of MDD. Increased gut permeability causes an influx of gut microbial components such as LPS, resulting in systemic inflammation and consequent neuroinflammation.

### Could microbiome-based therapies help?

Studies suggest they can. FMT given to patients to treat symptoms of IBS has been found to have beneficial secondary effects on symptoms of depression (Huang et al., 2019), while a meta-analysis that pooled the results from 34 clinical trials concluded that probiotics have modest beneficial effects on depression and anxiety (Liu et al., 2019). Two randomised, double-blind, placebo-controlled trials in this meta-analysis are explored in more detail. In the first study, 40 MDD patients were treated with a probiotic capsule (*Lactobacillus acidophilus* + *Lacticaseibacillus casei* + *Bifidobacterium bifidum*) or a placebo for 8 weeks. Significant improvements were seen in depressive symptoms, insulin resistance marker, and C-reactive protein in the probiotic group relative to controls (Akkasheh et al., 2016). In the second study, 81 MDD patients were given probiotics (*Lactobacillus helveticus* R0052 + *Bifidobacterium longum* R0175), prebiotics (galacto-oligosaccharide) or a placebo for 8 weeks, with symptoms of depression significantly improving only in the probiotic group relative to controls (Kazemi et al., 2019).

The latest research on LcS supported these observations; a 12-week open-label study of a LcS-fermented milk drink on patients with depression found improved depressive symptoms and sleep quality (Otaka et al., 2021). Another study revealed that 8-week of treatment with a LcS-fermented milk drink significantly attenuated the stress-induced rise in salivary cortisol in medical students under academic stress (Takada et al., 2016). These results suggest that some probiotic strains can modulate stress-induced activation of the HPA axis and the subsequent onset of depression.

In summary, the GM is likely involved in the pathophysiology of MDD via several pathways, and GM modulators, including probiotics and FMT, could be helpful adjunct therapies.

## Overcoming the brain barrier: a challenge for bacteria?

Implicating the GM in the pathophysiology of brain diseases and conditions requires that bacterial substances can access brain tissues. How this might occur was the topic reviewed by Prof. Roosmarijn Vandenbroucke from the Flanders Institute for Biotechnology (VIB) and Ghent University, Belgium.

The brain is protected from the peripheral circulation by central nervous system barriers, which include the blood–brain barrier (BBB) and the lesser-known blood-cerebral spinal fluid (CSF) barrier, which sits within the brain ventricles. Both barriers are characterised by being selectively permeable and having several parts to their structure, including a layer of epithelial cells in the case of the blood-CSF barrier and endothelial cells in the case of the BBB; both possessing tight junctions which regulate access. The choroid plexus epithelial cells at the blood-CSF barrier share similarities with those in the gut and have microvilli at their apical side, enhancing the surface area.

### Barrier functions

There is a difference in permeability between the two barriers since the capillaries which sit underneath the choroid plexus epithelial cells that form the blood-CSF barrier are fenestrated (ie. leaky). This means no tight junction proteins connect the choroid plexus endothelial cells to one another (Vandenbroucke, 2016). The purpose of the choroid plexus is to remove waste products from the brain, act as its gatekeeper, and make CSF, a soup of different molecules, including nutrients, neurotrophins, and growth factors. The choroid plexus epithelial cells are in very close contact with the endothelial cells of the capillaries inside the choroid plexus. This enables them to respond to triggers from the peripheral circulation, such as cytokines, and consequently relay these peripheral signals to the brain, but how does this process occur?

One answer is via extracellular vesicles, cell-derived nanoparticles that transfer biological cargoes between cells and can cross the brain barriers bi-directionally, giving them a powerful influence across the body. Extracellular vesicles may originate from the body’s cells or from bacteria, which potentially explains how the GM could have an impact on the brain. This was demonstrated in an animal experiment (Balusu et al., 2016b) where LPS was peripherally delivered, resulting in systemic inflammation and inflammation in brain cells. An extracellular vesicle inhibitor was then administered in the brain, which blocked the inflammatory signal to the brain, suggesting that extracellular vesicles act like a relay between the peripheral circulation and the brain. Animal studies suggest that a healthy GM is essential for the optimal development of the BBB since germ-free mice display increased BBB permeability compared with pathogen-free controls with normal GM (Braniste et al., 2014). There is also evidence that choroid plexus dysfunction via altered secretory, transport, and immune. Barrier function plays a central role in ageing and the risk of developing conditions such as Alzheimer’s disease (Balusu et al., 2016a). Hence, targeting the GM composition, or administrating SCFAs might have therapeutic potential.

### Bacterial extracellular vesicles and brain diseases

The discovery of extracellular vesicles, especially those derived from bacteria, has advanced understanding of how gut dysbiosis may influence the initiation and progression of chronic progressive brain conditions. One example is the association between *Helicobacter pylori*, a gastrointestinal pathogen found in around half of adults, and an enhanced risk of Alzheimer’s disease. It has been hypothesised that bacterial-derived EVs, called outer membrane vesicles (OMV), if derived from Gram-negative bacteria, can cross the brain barriers, and initiate pathogenic changes, such as neuroinflammation or beta-amyloid plaque deposits (Xie et al., 2022). This was studied by loading *H. pylori* OMV with cre enzyme and feeding these to tdTomato reporter mice, genetically engineered mice whose cells turn red when cre is taken up. This study showed an apparent increase in red astrocytes, confirming that *H. pylori* OMV had travelled from the gut to the brain, crossing the brain barriers (Xie et al., 2022).

The impact of this was investigated by feeding wild-type mice with *H. pylori*-derived OMV and studying the activity of cells in the brain (Xie et al., 2023). OMVs were found to overstimulate the microglia, leading to excessive synaptic pruning, evidenced by reduced dendrite length. Electrophysiological measurements then confirmed that *H. pylori*-derived OMV had detrimental effects on synaptic activity. To examine the clinical impact of OMV, a mouse model of Alzheimer’s disease was treated with OMV for 3 weeks. The findings confirmed a significant effect on plaque deposition with more plaques and a larger plaque area than control mice. Hence, *H. pylori* OMV can access the brain and potentially accelerate pathogenic changes associated with Alzheimer’s disease. At this stage, it is unclear how the OMV are crossing the blood-CSF barrier.

In summary, a functioning blood-CSF barrier requires the presence of a GM and is strengthened by SCFA-producing taxa. Recent research shows that *H. pylori* OMV can enter the brain and accelerate changes associated with Alzheimer’s disease, such as glial activation and plaque deposition.

## IBS: is it all between the ears?

This was the intriguing question asked by Prof. Francisco Guarner from the Teknon Medical Centre, Spain. Irritable bowel syndrome (IBS) is characterised by chronic, relapsing diarrhoea or constipation with no detectable cause. Bloating and pain are common symptoms (Lacy and Patel, 2017), often blamed on intestinal gas, but the symptoms could be due to heightened sensitivity to abdominal distention. This was demonstrated in an experiment (Barba et al., 2019) where patients who had reported reactions after eating lettuce were given an abdominal computer tomography scan before and after eating this trigger food. Average post-prandial girth increased by 35 mm, representing an 835 ml expansion of intra-abdominal volume, but only 40 ml of this was due to extra gas, which was within the normal range. It was concluded that patients felt bloated because consuming lettuce led to a conditioned response of diaphragm displacement, with computer tomography scans showing an average diaphragm descent of 7 ± 3 mm. Following behavioural training, patients reduced their anxiety-related response to trigger foods by learning to control their diaphragm movement.

### Is dietary restriction necessary for IBS?

The low FODMAP diet, which restricts poorly absorbed short-chain carbohydrates including fructose, lactose, polyols, fructans, and galacto-oligosaccharides, is a favoured treatment for IBS and resolves symptoms in 50–80 per cent of patients (Staudacher and Whelan, 2017). However, it entails short-to-medium term avoidance of certain foods, particularly plant-based foods, which may be neither convenient nor healthy for the patients. Hence, it may be better to employ cognitive behavioural therapy to condition a more positive response to trigger foods (Black et al., 2020). This has led to proposals that diet-induced symptoms in IBS are driven by dysregulation of the gut–brain axis since blinded interventions reveal similar increases in small bowel motility and colonic gas volume when IBS patients and healthy controls consume fructans (Wu et al., 2022).

Gut bacteria create intestinal gas by fermenting carbohydrates which begs the question: do IBS patients have a particular GM profile? In one study (Manichanh et al., 2014), patients complaining of flatus were compared with healthy controls before and after a 3-day challenge diet that was rich in plant foods. Even on the baseline “usual” diet, patients reported more abdominal symptoms and gas than controls, which worsened in both groups following the challenge diet. Changes in the GM of patients mirrored the increased symptoms, with *Bilophila wadsworthia* correlating with the increased volume of gas expelled. However, the GM of patients reduced in diversity and changed more radically in response to the challenge diet compared with the controls, which remained relatively stable. Hence, the GM of IBS patients, whether due to their habitual diets or other lifestyle factors, appears to be less adapted to digesting a plant-based diet and more adapted to digesting protein. This may lead to a predominance of gas-producing taxa.

### Implications for wider health

While it is difficult to differentiate people with and without gut dysbiosis simply by looking at their GM, there are associations between digestive symptoms and particular taxa. Pozuelo et al. (2015) found that patients with IBS had significantly lower microbial diversity and fewer microorganisms that produce butyrate and methane. Since these are responsible for disposing of hydrogen in the gut, their lower abundance in people with IBS could explain the excess of abdominal gas. *Prevotella* was more associated with healthier controls; interestingly, these taxa can digest vegetable matter. IBS is not the only condition characterised by microbial indicators since a study in 8,208 Dutch adults found that the GM of people with cancer, diabetes, cardiovascular disease, and neurological conditions share microbiome commonalities and could be differentiated from the GM of healthy people (Gacesa et al., 2022).

If the healthy GM profile favours those species adapted to ferment fibre-rich plant substrates, could a low FODMAP diet, which typically restricts these foods, drive unhelpful changes in the GM? This could be true, according to research that finds that a low FODMAP diet leads to atrophy of taxa adapted to digest vegetables (Halmos et al., 2015). Hence, alternative therapies are warranted to enable people with IBS to follow the recommended plant-rich diet for general health and disease prevention. Huaman et al. (2018) combined a Mediterranean (Med) diet with a prebiotic (galacto-oligosaccharide), which was tested against a low FODMAP diet in a randomised controlled trial (RCT). Similar reductions in gut symptoms were seen after 4 weeks on both diets, except flatus which was reduced only after the low FODMAP diet. However, some of these benefits were not sustained, as symptoms reappeared immediately after patients discontinued the low FODMAP diet. In contrast, the benefits of the prebiotic-Med diet combination persisted during the 2-week follow-up when patients returned to their habitual diets. In addition, the diets had opposite effects on *Bifidobacterium* sp., with a decline seen after the low FODMAP diet versus an increase after the prebiotic-Med diet.

In summary, as plant-based diets are now widely recommended for health and disease prevention, it is important that people with IBS are supported to eat these by employing behavioural strategies, which condition a positive response to trigger foods, rather than managing their symptoms with trigger food avoidance.

## Gut microbial diversity: one health and probiotics

Taking his cue from One Health – the European program which recognises the interconnectivity between the environment and human/animal health – Dr Olaf Larsen from Yakult Nederland and Vrije Universiteit, The Netherlands, discussed the role of microbial diversity, particularly of key taxa and guilds in promoting health.

The worldwide incidence of infectious diseases, including tuberculosis and measles, declined dramatically during 1950–2000 against a backdrop of rising autoimmune disorders, such as type 1 diabetes (T1D), Crohn’s disease, and asthma (Bach, 2002). This trend continues in a more recent analysis (Larsen et al., 2022). In particular, T1D incidence has risen steadily in Europe and the US over the past 40 years. However, as demonstrated by the SARS epidemics and the SARS-Covid-19 pandemic, infectious diseases are far from being eradicated.

### Old friends

While the fall in infections is understandable, given vaccines and improved hygiene standards, the reason for the rise in autoimmune problems is less clear and may be related to the health of our microbiota. One theory is that humans, especially in early childhood, have limited exposure to beneficial microbes from food and the environment – referred to as “old friends” – which leads to an overreactive immune system with the propensity to attack the body’s own tissues as well as overacting to harmless microorganisms or antigens. Indeed, studies show that diminished exposure to microorganisms in early life correlates with an increased risk of atopic diseases (Von Mutius et al., 2000).

A deterioration in GM balance in Western countries has been cited as a reason for their greater burden of Covid-related mortality and higher rates of autoimmune and chronic non-communicable conditions. GM diversity correlates with risk (Dhar and Mohanty, 2020) and severity of Covid-19 (Yeoh et al., 2021). It may also influence the development of metabolic syndrome (Fan and Pedersen, 2021) which increased in prevalence from approximately 30 to 40 per cent of the US adult population during 2000–2018, emphasising the immense scale of this issue (Larsen et al., 2022). Completing the circle of disease risk, SARS-Covid-19 infection has been found to exacerbate metabolic disease (le Roux, 2021).

All of this indicates a need for Western populations to improve their exposure to “old friends” and regain microbiota eubiosis – considered to be a state of balance in the GM between beneficial and harmful bacteria, which is normally associated with a disease-free host. The human gut loses a proportion of the conserved microbiome with each successive generation, possibly related to incomplete maternal-child transmission (due to Caesarean births and lower than ideal breast-feeding rates) and excessive antibiotic use, which has remained relatively stable despite concerns about antibiotic resistance (Blaser and Falkow, 2009). New exposures do not compensate for this decline in beneficial bacteria since society has adopted unhelpful practices of indoor living and diets lacking in fermented foods (FF). If these ecosystem losses continue, a catastrophic collapse in the GM is hypothesised leading to abrupt and possibly irreversible shifts between alternative ecosystem states (Larsen and van de Burgwal, 2021). Increasing GM diversity increases functionality, for example, SCFA production, but only if the right species are introduced. If the wrong diet and lifestyle are adopted, less favourable species could thrive, reducing ecosystem resilience and creating functional redundancy.

### Keystone taxa and guilds

A balanced GM includes keystone (core) taxa and guilds. Keystone taxa are: “highly connected taxa that individually or in a guild exert a considerable influence on microbiome structure and functioning, irrespective of their abundance, [hence] their removal can cause a dramatic shift in microbiome structure and functioning.” (Banerjee et al., 2018). Guilds are small ecosystems where 2–10 taxa work together as coherent functional units or exploit the same type of resources (Maurice and Turnbaugh, 2018).

The absence of specific guilds has been linked with a greater risk of autoimmune conditions and certain metabolic diseases, but these guilds can be restored with the right interventions, which may include FMT, probiotics, or dietary fibres. In one RCT in people with type 2 diabetes, diets high in fibre promoted SCFA-producing strains at the expense of strains that produced potentially detrimental compounds such as indole and hydrogen sulphide (Zhao et al., 2018). These GM changes in the high fibre group were associated with improved haemoglobin A1c levels. At present, the evidence is insufficient to determine whether single-strain or multistrain probiotics are more effective at restoring eubiosis (McFarland, 2021) although the theoretical research suggests a higher diversity in microbial guilds leads to a more efficient system. Hence, the choice of an appropriate probiotic should be based not on the number of strains in the product but on evidence-based efficacy trials. There is also an issue with non-responders which implies that a personalised approach is needed to determine the correct keystone taxa and guilds.

In summary, to avoid the risk of catastrophic collapse in the GM, we need to take a One Health approach to promote microbiota eubiosis. This includes greater biodiversity and exposure to “old friends,” appropriate substrates from high-fibre and plant-rich diets, as well as limiting antibiotic use and excessive hygiene.

## Opportunities relating to functional foods

One source of “old friends” is traditional FF, according to Prof. Paul Cotter, from the Teagasc Food Research Centre and APC Microbiome Ireland, who reviewed some recent research on this topic.

FF are “foods made through desired microbial growth and enzymatic conversions of food components” (Marco et al., 2021). Examples include kefir, sourdough bread, yoghurt, kimchi, and kombucha. The different microbes used to make FF determine the fermentation process, flavour, nutrients/bioactive compounds, and potential health benefits, including nutritive alteration of the ingredients, presence of bioactive compounds that affect intestinal and systemic function or modulation of the immune system. However, not all FF work as probiotics and referring to FF microorganisms as probiotics is misleading unless backed by evidence from human studies.

Used as a means to preserve foods, FF have a long history of use in nations around the world (Gänzle, 2022; Jimenez et al., 2022). The expansion of modern research techniques has helped investigate the microbiota of FF, highlighting differences across foods and, indeed, different versions of the same food type. This inherent variability has complicated standardisation, an issue further complicated by different standards and regulations between countries (Mukherjee et al., 2022). As an example, the term “kefir” is reserved for dairy in some jurisdictions, eg. Germany, and cannot be applied to water kefirs.

### Fermented food research

A global initiative was set up to apply shotgun metagenomic sequencing to a diverse range of FF, eventually sourcing 58 international artisan products (Leech et al., 2020). Food type, for example, dairy, brine- or sugar-based, was the primary driver of microbial composition, and foods within these clusters had more similar microbiomes than those from other clusters. Several FF did not fit with any cluster, including coconut kefir and soya-based foods for which there are relatively little data. Multiple potentially novel microbial species were identified, which could represent untapped functionality resources.

Further work (Pasolli et al., 2020) has mapped lactic acid bacteria (LAB) species found in FF with those present in the human GM, finding that, for some species, closely related LAB strains occur in both food and gut environments. This provides new evidence that FF can be a source of LAB for the gut microbiome. The next phase will look at African FF as these have been under-researched. Africa offers a wealth of FF examples that contain microbes differing significantly from those found in FF from other continents.

Microbiome Applications for Sustainable food systems through Technologies and Enterprise (MASTER) is a new initiative that applies analytical techniques to FF typically used to study the human GM. One MASTER study (Cotter, personal communication) found specific clusters of microbial genes associated with colonisation, gut survival, modulation pathways, and human health within FF microbes. Indeed, FF contained significantly more health-associated gene clusters than non-fermented substrates, indicating the transformative influence of adding microbes to foods. The work could be used to identify which FF are worth testing in human clinical trials.

### The example of water and milk kefir

Kefir grains contain a consortium of bacteria and yeasts, although the specific microorganisms in water and milk kefir grains are very different. Water kefir is a fermented beverage made by inoculating water kefir grains into a sugar (sucrose)-rich solution supplemented with fruits. Often made in a household setting, the mixture is left to stand at room temperature for 1 to 3 days, after which the grains are filtered out to obtain the final drink. A recent study (Mortensen, unpublished data) sourced water kefir grains from around the globe and fermented them with the same substrate. Heat maps based on microbial taxonomy revealed differences in α-diversity across countries and at least 10 clusters of microbial communities which may be important for flavour, shelf life, or health. This work could help define international standards for water kefirs, which tend to differ from country to country regarding their microbiome.

Milk kefir is made by fermenting milk with milk kefir grains. Research has identified specific microbes linked to volatile compounds which could help develop optimal flavour profiles for new products, for example adding *Lactobacillus kefiranofaciens* NCFB 2797 to increase fruitiness (Walsh et al., 2016). This work is being expanded to 64 international milk kefir samples to determine theoretically which microbes could indicate potential health attributes. This is important as, while milk kefir has been linked with several health benefits including cholesterol reduction and antimicrobial activity, the quality of evidence is often poor (Bourrie et al., 2016). Notably, some animal studies evaluating the impact of kefir on obesity, dyslipidemia, and metabolic diseases suggest that the health-promoting attributes of kefir depend on specific microbes, which could explain why some kefirs do not produce any health effects (Bourrie et al., 2020). Indeed, a study to deconstruct the microbes in artisan kefirs found that *Lactobacillus* and yeast were essential components for lowering plasma cholesterol in mice (Bourrie et al., 2021).

Ultimately, understanding which microbes in FF are important for health could help inform standards for commercial products and may eventually lead to population recommendations for specific microorganisms to be consumed through the diet.

## Living foods: safe salvation for health

Continuing the theme of FF, Prof. Lorenzo Morelli, from the Catholic University of the Sacred Heart, Italy, described how modern research techniques can improve the understanding of traditional production methods.

Since around 7000 BC, humans have preserved protein-rich foods using different methods, including salt, smoke, and fermentation. The term fermentation comes from the Latin verb “fervere,” which means “to boil,” possibly referring to the bubbles seen when liquids are fermented. An example of a traditional FF is Parmesan cheese which is still made only with raw milk in copper pots using the previous day’s culture – called “back slopping.” Commercially available bacteria cultures are not permitted and the cheese must be ripened for more than a year for reasons of safety. There is good genetic evidence that these traditional methods have selected a sub-population of LAB whose chromosomes are adapted to making Parmesan cheese since they can grow at 51 °C, which is unusually high for such bacteria.

### New methods to solve old problems

However, a weak point of traditional back slopping is the undefined age and viability of the bacterial cells, given that cheese-making requires the correct balance of lactic acid and viable cells. Older bacteria produce too much lactic acid which eventually kills the culture. Uncertainty can also be introduced by raw milk, whose composition and bacterial profile are influenced by different seasons and pastures. Newer research technologies can be used to address these traditional problems. In one study (Bellassi et al., 2021), researchers used metabolomics and genomics to discriminate between milk produced by cows fed hay and milk from cows fed hay and fresh vegetables.

The bacteria used to make FF are multifunctional, transforming raw ingredients’ aroma, flavour, taste, and durability. It has been found that sourdough cultures are essential for flavour and leavening and act as natural preservatives (Bourdichon et al., 2021). Biopreservation refers to enhanced food safety and extended shelf life of foods by indigenous and/or intentionally added microbiota, inhibiting the growth of pathogenic and spoilage organisms due to microbiological competition and production of antimicrobial metabolites (Shi and Maktabdar, 2022). This is an important attribute as consumers want foods to have a longer shelf life yet remain concerned about chemical preservatives and plastic packaging. There is a potential role for LAB against fungal spoilage of foods (Siedler et al., 2019), as demonstrated by an experiment that found that breads inoculated with mould were better preserved after 7 days when made with LAB compared with regular yeast. Modern techniques could be used to leverage these hitherto unknown benefits of cultures. The antimicrobial characteristics of several microorganisms are already recognised by generally recognised as safe (GRAS).

### Human impact

LAB also interact with our bodies, as first recognised by Russian zoologist and Nobel laureate, Élie Metchnikoff (Mackowiak, 2013), who hypothesised in the early twentieth century that “intestinal putrefaction” shortens life but that lactic acid could be an antidote. This led him to be the earliest advocate of LAB as therapeutic agents and he is often considered the “father” of probiotics. While Metchnikoff’s original experiments could be described as hazardous – for example, injecting himself with pathogens or feeding lactic acid to volunteers – he went on to advocate the use of LAB in FF, stating in 1907: “*Dependence of the intestinal microbes on the food makes it possible to adopt measures to modify the flora in our bodies and to replace the harmful microbes by useful microbes.*”

Metchnikoff incorrectly assumed that colonic bacteria could be modulated using supplemental lactic acid. Still, it is reasonable to assume that the GM could be influenced by a range of LAB by-products found in FF, including bioactive peptides (Ali et al., 2022). These have been associated with anti-hypertensive, angiotensin-converting enzyme (ACE) inhibitory, antioxidant, anti-inflammatory, and immunomodulatory effects, which could deliver health benefits (Beltrán-Barrientos et al., 2016; Raveschot et al., 2018). Bioactive peptides may also improve mineral bioavailability (Tenenbaum et al., 2022), which could support healthy ageing and the prevention of osteoporosis. Since the neurotransmitter γ-aminobutyric acid (GABA) is one of the by-products of LAB metabolism, it has been hypothesised that FF could influence the brain. The potential anti-hypertensive effects of reduced sodium sourdough, made with *Levilactobacillus brevis* CECT 8183, were investigated in a laboratory study (Peñas et al., 2015). The results showed significantly increased total antioxidant activity, GABA levels, and ACE inhibitory effects compared with the control bread, suggesting that innovative breads could be developed to reduce blood pressure.

Hence in summary, while there is a long history of humans using bacteria to preserve nutrients through fermentation, their interactions in our bodies and potential impact on health are only beginning to be understood.

## Living drugs: a solution with many benefits

This narrative was continued by Prof. Stephan C. Bischoff, from the University of Hohenheim in Stuttgart, Germany, who described how FF evolved first into functional foods and supplement products, then medical applications. These require different approaches to safety assessment, regulation and methodologies to establish evidence of efficacy. This is because the purpose of probiotics has evolved from health maintenance to the prevention, management, or treatment of diseases and abnormal conditions.

Oral microbiota therapy can include probiotics, prebiotics, and postbiotics; the latter being inanimate microorganisms and/or their components that confer a health benefit on the host (Salminen et al., 2021). For probiotic medical trials, it is crucial to consider strain, dosage, target population, disease type, and progression. Understanding mechanisms is also vital to support medical claims and ensure that the right probiotics are targeted at the right population of patients (Daliri et al., 2021). Given recent advances in knowledge, relevant pathways of action include the gut–brain axis and the gut–liver axis, with the potential for probiotics to modulate a range of metabolic, inflammatory, and neurological conditions. So, where is the evidence currently?

### Respiratory tract infections

Cochrane reviews are a gold standard of independent systematic review and meta-analysis (SRMA). In one of these, probiotics were found to lower the incidence but not the duration of respiratory tract infection (RTI) (OR 0.58; 95% CI 0.36–0.92) and reduced antibiotic prescriptions (0.67; 95% CI 0.45–0.98) (Hao et al., 2011). These conclusions were confirmed in updated reviews of studies on adults and children (Hao et al., 2015; Quick, 2015). Other SRMAs have concluded that probiotics and prebiotics effectively improved response to the influenza vaccine (Lei et al., 2017), while fermented dairy products protected against RTI (Rashidi et al., 2021).

However, one issue with SRMAs is the heterogeneous approach to probiotic strains, that is, dosage and duration of the administration, which can create inconsistencies that make null conclusions more likely. Another issue is that SRMAs can be based on several small pilot trials subject to publication bias. Hence, there is a need to consider well-conducted large RCTs, of which several now exist:

- A 6-week trial of three probiotics on common cold symptoms in 581 college students found that *B. bifidum* increased illness-free days (Langkamp-Henken et al., 2015).
- A 6-month trial in 171 children found that a probiotic plus vitamin C reduced coughing, absenteeism, and antibiotic usage (Garaiova et al., 2021).
- Two 12-week trials of fermented milk with LcS found prevention of the common cold and influenza in 96 office workers (Shida et al., 2017), and reduced risk of acute upper RTI in 1003 children (Mai et al., 2021).

Moving to the hospital environment, the severe condition of ventilator-associated pneumonia is a common issue for intensive care patients. Here, too, SRMAs have confirmed that probiotics have a therapeutic role in this condition, as there is robust evidence for a 30 per cent reduction in risk (Bo et al., 2014; Ji et al., 2021; Sharif et al., 2022). A large RCT backs this using a 4-strain preparation (*L. acidophilus*, *Lactiplantibacillus plantarum*, *Bifidobacterium animalis* subsp. *lactis,* and *Saccharomyces cerevisiae* var boulardii) in 112 trauma patients (Tsilika et al., 2022). However, another large RCT (*n* = 2,653) found no significant benefit *of Lacticaseibacillus rhamnosus* GG for ventilator-associated pneumonia (Johnstone et al., 2021).

### Gastro-intestinal disorders

A major indication for probiotics is antibiotic-associated diarrhoea. The evidence for *S. cerevisiae* var boulardii and *Lactoballicus* sp. is so well established, with a risk reduction of more than 50 per cent (Szajewska and Kołodziej, 2015a, 2015b) that further data are unnecessary.

Probiotics are also recommended in the German IBS guidelines since few effective drug treatments exist for this condition (Layer et al., 2021). However, the opposite is true for inflammatory bowel diseases, such as ulcerative colitis and Crohn’s disease, where probiotics offer weak beneficial effects that are inferior to drugs (Kaur et al., 2020). Small intestine bacterial overload results from gut dysbiosis and is characterised by bloating, pain, and post-prandial diarrhoea. A SRMA by Zhong et al. (2017) found that probiotics could not prevent small intestine bacterial overload but lowered gut hydrogen levels and improved treatment efficacy, including for abdominal pain. German guidelines indicate that it is best practice to use probiotics alongside antibiotics and a low FODMAP diet (Layer et al., 2021).

In medicine, probiotics are most effective for RTI and gastrointestinal conditions. In the future, probiotics could treat other types of conditions such as metabolic syndrome, obesity, and neurological diseases but, to do this, new probiotics need to be discovered and tested in clinical trials. In a recent trial (Gutiérrez-Castrellón et al., 2022) a new patented 4-strain probiotic improved remission rates and viral load in patients with SARS-Covid-19. Further research and product development are required to deliver the advantages of living drugs to all parts of the body.

## Safety of microorganisms used as probiotics

Before being included in the food system, microorganisms must be risk assessed to ensure consumer safety. Is the current system fit for this purpose? This was discussed by Prof. Pier-Sandro Cocconcelli from the Università Cattolica del Sacro Cuore, Italy, who identified four trends in risk assessment (RA).

Microorganisms are deliberately introduced into the food chain to assist food production (eg. to create FF) and to benefit animal and human health. RA involves hazard identification and characterisation, exposure assessment, and risk characterisation, but this system was designed with pathogens, not probiotics, in mind. Hence, adjustment is needed to enable the system to provide adequate assessment, for example, using dosage data from intervention studies rather than population exposure.

### Trend #1: the process of RA is rapidly evolving

Guidance on regulating microorganisms in food and feed has been rapidly evolving in Europe since 2005 due to the evolution of methodologies which have become increasingly complex since the advent of genomics.

### Trend #2: increased complexity of microbial RA

The RA system for microorganisms combines taxonomy, genomics, a qualified presumption of safety (QPS), AMR, virulence, and end-use. QPS is a fast-track approach that reduces unnecessary extensive safety testing by utilising the body of knowledge on the species plus a safety decision tree. It differs from the US system of GRAS, which is generally limited to a specific application made following a safety assessment (Franz et al., 2011).

More than 100 microorganisms have been granted QPS status in Europe, but their evidence is still updated bi-annually to ensure safety. For new microorganisms, the decision tree is followed and if QPS is not given, a full safety assessment is required. Even for QPS microbes, evidence of acquired AMR means that no approval will be given since the food system should not add to the burden of AMR and enable these genes to be mobilised in the human or animal gut. In contrast, intrinsic AMR is not considered a safety concern if this is inherent to wild-type bacterial species.

### Trend #3: genomics is fundamentally changing RA approaches

Some microorganisms have multiple characteristics ranging from pathogen to food culture, which taxonomy alone does not recognise; hence, genomic methods are needed. One example is *Enterococcus faecium* which can be a pathogenic, commensal, food culture, or probiotic organism, depending on the clade. While EFSA has produced guidance on genomics (European Food Safety Authority (EFSA), 2021), it refers to methods rather than purpose. In contrast, microbial RA is concerned with identification, genetic modification, and finding AMR genes, which suggests that the guidance on genomics needs updating.

Genomic techniques provide precise information on microbial phylogenesis but add complexity, making combining old and new data harder. In the case of *E. faecium,* gene sequencing can enable specific AMR genes to be identified. However, it can also overturn previous taxonomy as a study (Belloso Daza et al., 2021) concluded that clade B of *E. faecium* should be reassigned as *Enterococcus lactis.* Yet, while genomics may be suitable for identification, it still cannot tell us if microorganisms are safe. To do this, RA requires phenotypic testing based on determining a minimum inhibitory concentration of the potentially resistant gene and whole-genome sequencing to search for known AMR genes. In the example of *E. faecium,* whole-gene sequencing found mobilizable AMR genes in a sample taken from ready-to-eat sausages (Belloso Daza et al., 2022), highlighting the need for constant vigilance.

Yet, there remain shortcomings in using genomics to determine pathogenicity since genes for successful gut colonisation could act to promote virulence in a pathogen or survivability in a probiotic. Also, the definition and application of “intrinsic resistance” are not absolute, and there is a non-alignment between international regulatory bodies. Hence, an evolving approach to RA is needed.

### Trend #4: new products and applications

This impacts RA because it extends the continuum from natural to synthetic microorganisms. Synthetic biology is the application of science, technology, and engineering to facilitate and accelerate the design, manufacture, and/or modification of genetic materials in living organisms (SCENIHR et al., 2014). As new microorganisms could be potentially indistinguishable from non-genetically modified versions, RA should be based on the nature of the final strain and not on the methodology used to get there. The EU is considering how to regulate this area since genetically modified microorganisms are already present in non-EU markets. Another consideration is the RA of non-viable cells used in the food supply, such as postbiotics, which could be treated like biomasses or novel foods.

In summary, the regulatory system is still evolving to ensure proper RA of potentially useful microorganisms, aided by advancements in methodologies.

## The importance of the responder/non-responder issue for clinical trials

RA and authorization of health claims depend on high-quality evidence. Yet, the gold standard RCT may not be the most appropriate for nutrition research, including trials of probiotics, argues Prof. Robert Jan Brummer from Örebro University, Sweden.

In the hierarchy of medical evidence, the RCT is near the top, only surpassed by systematic reviews and meta-analyses of RCTs. While these types of studies undoubtedly work for medicine where drug compounds are standardised, relatively constant, and produce a large signal-to-noise ratio (ie. the effect of the intervention compared with the effect of interpersonal variations), they may not be appropriate for other health interventions which are not standardised, eg. because they are natural foods or ingredients, or where subtle changes in health are seen in the long term. Hence, the RCT model only works effectively to provide evidence of efficacy where certain assumptions can be made ie:

- External validity – being able to generalise the findings of RCTs to a defined population;
- Independence of effects – where the observed effect is most likely due to the intervention and not a confounding variable;
- Adequate characterisation of the intervention and placebo (Zeilstra et al., 2018).

These assumptions may not always be valid for nutritional interventions, such as dietary interventions or probiotics, which can often yield inconsistent results in RCTs, which are then amplified in meta-analyses.

### External validity

To be clinically useful, nutritional interventions must work in a definable group of people (age, sex, health status, nutritional status) in a particular public health or hospital setting. Lack of external validity is one explanation for the widespread underuse in the routine practice of many treatments that were shown beneficial in trials and are recommended in guidelines (Rothwell, 2006). Inter-individual variation in participant response is a barrier to external validity because, unlike pharmaceuticals, nutritional interventions often have subtle effects which can be overwhelmed by the background “noise” created by many individual variations in clinical response. A larger sample size does not help since this often increases the heterogeneity of the study population and inter-individual variation. One example is a hypertensive drug which would be expected to deliver a fall in systolic blood pressure of 10–15 mmHg (Paz et al., 2016), considerably greater in magnitude than the anticipated 4 mmHg fall from a 4 g reduction in salt intake (He et al., 2013) which would be a significant dietary shift for the target population. Hence, in the presence of non-compliance and intra-individual variation, the dietary intervention must work harder than a pharmaceutical treatment to achieve a statistically significant result.

### Independence of effects

It would be illogical to combine all brands of hypertensive drugs into one RCT. Yet, trials of probiotics often mix species and strains into one intervention, reducing the chances of a clear, unambiguous result. This is then compounded by systematic reviews and meta-analyses that pool studies using various strains. Different strains of probiotics have different clinical effects, making it necessary to understand the mode of action to select the correct outcome variable and patient group. It is also essential to control the potential for bias, particularly from the rest of the diet.

### Adequate characterisation

It is a fundamental error to assume that probiotics are standardised simply because they can be put into capsules like drugs. Probiotics are living organisms that evolve once they reach the recipient’s colon, depending on the available substrates provided by the diet, eg. the amount and types of fermentable carbohydrates and proteins. This means that the same product does not deliver the same treatment in every recipient; thus, in the case of probiotics, the idea that the treatment is well-defined may be questionable (Zeilstra et al., 2018).

### Way forward

Three concepts may be considered to address the issues of inter- and intra-individual variation. Firstly, by considering responsive nutrition, which aims to target interventions by identifying likely responders through machine learning analysis of health, genetic, drug, and dietary data. This could create a phenotype for optimal responsiveness, which could help target probiotic interventions to those most likely to respond. Responsive nutrition differs from personal nutrition. The latter focuses on providing the best dietary intervention on an individual basis. Secondly, by trying to limit intra-individual variation as far as possible. This could be done by conducting many trials, on fewer people with a stable background pattern of the primary outcome measure, rather than one trial on many people with unspecified intra-individual variation (Larsen et al., 2020). Thirdly, by using surrogate biomarkers which show short-term changes predictive of a health effect instead of using medium-term disease markers which other lifestyle factors may influence. One example is functional brain imaging which, in a 4-week RCT of probiotics (Rode et al., 2022), demonstrated significant changes in brain morphology and resting-state brain function linked to stress management of the brain.

In summary, non-response and intra-individual variation hamper a clear understanding of the efficacy of probiotics, and we need to look beyond the classic RCT design to overcome this challenge.

## Development of the infant microbiota

Turning from foods back to the human body, Prof. Christoph Lacroix, from ETH Zurich, Switzerland, described the acquisition of the microbiome in infanthood and discussed how different lifestyle and environmental factors can influence which taxa thrive, hence, which functions are expressed.

From the sterile environment of the womb, the infant’s gut is rapidly colonised by pioneer microorganisms (Khan et al., 2015), evolving in terms of taxa and diversity over the first few years. This remains relatively stable until old age, when diversity declines. Modern techniques like metagenomics enable us to look at microbial function over the life course, which is more important than taxonomy.

### GM acquisition

Initially dominated by LAB, the infant gut microbiome changes most rapidly between the ages of 1 and 6 months with the cessation of breast-feeding, rather than the introduction of solid food, correlating with maturation into an adult-type microbiota (Bäckhed et al., 2015). Building on this research, Roswall et al. (2021) conducted a longitudinal cohort of 471 healthy Swedish children to track the development of the GM from birth to 5 years, noting four discrete trajectories for different microbes. The greatest changes occurred in the first year of life, and by the age of 3–5 years, the child GM was closest to that of adults, although still evolving.

Roswall et al. (2021) identified four major trajectories for individual genera in the developing GM of infants and young children, with some genera peaking at 4–12 months, others increasing rapidly between 4–12 months before stabilising by 3 years, and a final group increasing in relative abundance after 12 months and continuing to increase until 5 years. These shifts were linked to the cessation of breast feeding, the introduction of solids, increased socialisation outside the family and increased diet diversity.

Both vertical (from the mother) and horizontal (from the birth environment) transmission determine which pioneer species colonise the post-natal gut. Factors include maternal diet and health, vaginal versus Caesarean birth, skin-to-skin contact, breast or bottle feeding, and antibiotic use (Marques et al., 2010). Molecular methods have revealed the presence of more microorganisms in human milk than previously believed, such as skin bacteria, Bacteroidota phylum, and clostridia (Selma-Royo et al., 2022). Indeed, the bacterial diversity of human milk may even exceed that of neonatal faeces (Jost et al., 2013). However, this could be explained by different population densities and structures and the limited resolution of the sequencing methods. There is also evidence of bacterial translocation through the entero-mammary pathway since similar strict anaerobe species and strains have been found in maternal faeces, breast milk, and infant faeces (Perez et al., 2007).

### Beneficial role of microbes

A comprehensive study tracked the impact of breastfeeding on GM changes in seven healthy neonates aged 4 to 30 days (Jost et al., 2012). Neonate faeces were dominated either by *Bifidobacterium* or *Bacteroides* sp. Strict anaerobes outnumbered facultative anaerobes within the first days, which was earlier than assumed, but major adult-type butyrate producers, such as *Roseburia* and *Faecalibacterium*, were not detected. While most infant gut bacteria are lactate producers from the main dietary carbohydrate lactose, some species must metabolise lactate, potentially toxic if allowed to accumulate, mainly into propionate (Chassard et al., 2014). Sulphate-reducing bacteria can remove hydrogen, a secondary metabolite produced by different taxa of the infant gut such as clostridia and *Veillonella* that may be linked to bloating and colic.

Such findings have led to the hypothesis that infants with colic may have more hydrogen-producing bacteria and/or fewer bacteria that can metabolise lactate and hydrogen. This was demonstrated in a 2-year prospective cohort study of 40 infants, including 8 with colic, which also found that peak lactate production occurred when infants were 2–3 months (Pham et al., 2017). Further research revealed a switch between the lactate-utilizer, hydrogen-producer *Veillonella* in the first year of life to the lactate-utilizer butyrate-producer, *Anaerobutyricum hallii*, in the second year of life, which was associated with weaning (Pham et al., 2022).

This was tested further in a gnotobiotic model (Rocha Martin et al., 2022) where rats were inoculated with faeces from healthy infants or those with colic. After milk formula feeding, rats with colic-associated microbiota produced significantly more hydrogen in faeces and had a higher abundance of *Veillonella* than healthy controls. Supplementation of the lactate-utilizer and propionate-producer *Cutibacterium avidum* P279 to rats with the colic-associated microbiota reduced gut hydrogen levels compared with animals receiving a placebo. The results confirm the benefit of cross-feeding between bacteria in the infant gut and suggest that targeted probiotics could help to manage colic.

In summary, these studies suggest a broad window of opportunity for dietary interventions tailored to support the evolving infant GM. A good example is the promotion of taxa involved in lactate and hydrogen cross-feeding to help address infant colic. However, more research is needed to understand better the mechanisms and functions of the infant GM, particularly from low- and middle-income countries.

## Microbiota composition from 1 till 100

Beyond infanthood, the GM continues to change, with implications for long-term health, as discussed by Prof. Gaspar Pérez Martínez from the Institute of Agrochemistry and Food Technology (CSIC), Spain.

### The microbiome clock

While the GM of infants and adults differ in species, diversity, and functionality, a quantitative theory of intestinal ageing remains elusive because there are no recognised step changes in GM during adulthood. Some older adults have a GM similar to younger people, and there is an overlap between clusters of signature species linked to decades of life.

In a study of 367 healthy Japanese volunteers (Odamaki et al., 2016) from infanthood to very old age, bifidobacteria dominated in early life, but the relative abundance of Actinomycetota (formerly the Actinobacteria) substantially declined after weaning and was progressively replaced by Bacillota (formerly the Firmicutes). A further change occurred around 70 years when increases were seen in the relative abundance of Bacteroidota and Pseudomonadota while Bacillota declined. Using samples from 1,165 adults, a machine learning model could predict a healthy person’s age from their GM to an accuracy of fewer than 6 years. However, this did not work for patients with T1D who exhibited microbiome age acceleration (Galkin et al., 2020). In a different study (Bian et al., 2017) with 1,000 healthy Chinese volunteers, GM patterns showed remarkable similarities between healthy aged and younger adults for overall GM composition, a fact observed in previous studies (Odamaki et al., 2016). In this case, health was a better predictor of GM ageing than years of life. Interestingly, this study also revealed a stable diversity across all age categories, with a shift in GM profiles around 19–24 years of age which could reflect changes in hormones or lifestyles, eg. going to university or the army.

### Factors affecting GM composition across life

Five factors were outlined: environment, diet, genetics, antibiotics, and health.

#### Environment

Children exposed to less urbanised environments have a lower risk of autoimmune conditions. Studies in Finnish and German children (Kirjavainen et al., 2019) found a reduced incidence of asthma in farm-raised children, with the indoor dust of farmhouses having a lower abundance of Streptococcaceae. Asthma risk in children who did not live on farms decreased as their home microbiota composition became more like farm homes. Studies on tribal people have found a distinct and far richer GM diversity compared to industrialised populations (Clemente et al., 2015; Conteville et al., 2019), which could reflect the absence of antibiotics and differences in physical activity, diet, and exposure to outdoor microorganisms. People who exercise have a greater alpha diversity than sedentary people but few differences in taxa. The largest difference is in the metabolomics profile since regular exercisers have higher faecal SCFAs and harbour a greater proportion of phyla that break down carbohydrates, probably reflecting their habitual diets.

#### Diet

The GM responds to diet as it determines available substrates. A multi-centre metagenomics study (Arumugam et al., 2011) found three distinct clusters of GM composition associated with substrates rather than nationality. Subsequent studies collapsed these into two distinctive groups correlated with animal fat consumption: protein and simple sugars (*Bacteroides* group) or vegetables, complex carbohydrates, and fibre (*Prevotella* group). This was seen in practice when the GM was studied in people with different diets (De Filippis et al., 2016). Prevotellaceae were more abundant with plant-based diets, while Bacteroidota were more abundant in vegans and vegetarians than in omnivores. However, higher faecal SCFAs were seen with high dietary compliance, even in omnivores, when split by adherence to the Med diet. Changing the diet from meat-based to vegetarian, or vice-versa, can alter the GM, but only while the diet is maintained. Habitual vegetarians return more quickly to their baseline GM after resuming their usual diets (David et al., 2014). Consuming a functional drink based on *Cyperus esculentus L.* (tiger nuts) also shifted the GM pattern towards SCFA producers, but this depended on the baseline microbiome of each individual (Selma-Royo et al., 2022).

#### Genetics

A study of UK twins (Goodrich et al., 2016) uncovered familial hereditary lineages with greater similarities within the Ruminococcaceae and Lachnospiraceae families for monozygotic compared to dizygotic twins. An analysis of faecal samples from 71 individuals found that the diversity and composition of bifidobacteria were strongly associated with the histo-blood group ABH secretor/non-secretor status, which appears to be one of the host genetic determinants for GM composition (Wacklin et al., 2011).

#### Antibiotics

While having an overall positive influence on human health, antibiotics nevertheless inflict ecological disaster on the GM, wiping out helpful species alongside pathogens. The GM does regrow but typically does not achieve the same balance of species, particularly in people taking repeated antibiotic courses. Some individuals never recover their baseline GM (Chng et al., 2020). A SRMA (Duong et al., 2022) of observational studies found an increased long-term risk of auto-immune conditions and obesity in children given multiple antibiotic courses.

#### Health

Certain conditions have an impact on the GM. Coeliac disease changes the balance of GM species and increases diversity, while the time window between seroconversion and T1D in genetically susceptible children is characterised by reduced alpha diversity and a higher prevalence of species linked to inflammation (Kostic et al., 2015). These observations fit with the broader theory of gut dysbiosis affecting the aetiology of several chronic diseases, which could also be bi-directional, as demonstrated by the finding that sepsis induces low-grade inflammation and oxidative stress in the gut via such as TNF-α and interleukin-1β. This adversely changes GM balance since Reactive Oxygen Species have selective antibacterial effects (Cernada et al., 2016). At the other end of the age spectrum, there are associations between GM changes and the initiation of immunosenescence (Candore et al., 2008).

## GM changes in the young and old

Continuing the theme of looking at society’s oldest people, Prof. Patrizia Brigidi, from the University of Bologna, Italy, discussed the GM of centenarians using data on individuals from four distinct age groups (young, elderly, centenarians, and semi-supercentenarians) living in the same geographical area of Italy (Biagi et al., 2016).

Age is a key variable that impacts GM composition and function and represents an adaptive trajectory across the human lifecycle (Rampelli et al., 2020). GM changes provide the host with ecological services calibrated to each stage of life. For example, the relative importance of vitamin biosynthesis, fermentation, RNA degradation, and bile salt metabolism varies with age (Lynch and Pedersen, 2016). In particular, age-related changes impact on the GM composition and its crosstalk with the host, nurturing inflammageing, a chronic low-grade inflammatory status characteristic of advanced age, immunosenescence and metabolic disorders. These include changes in lifestyle, nutritional behaviour, prescribed drug use, and gut physiology and functionality such as reduced intestinal motility and increased intestinal permeability.

Healthy semi-super centenarians, aged 105–109 years, represent a good model for studying healthy ageing as they have survived for 20 years longer than their demographic cohort and somehow escaped the major chronic age-related disorders and causes of mortality. The GM of this group was compared with three other sub-groups with mean ages of 100, 72.5, and 30.5 years based on 16S rRNA amplicon sequencing analyses (Biagi et al., 2016). The GM composition in the youngest and oldest groups could be clearly differentiated, with the middle age groups having some overlap as well as age-related biodiversity decline. A core of highly prevalent bacteria, mostly belonging to Ruminococcaceae, Lachnospiraceae, and Bacteroidaceae families was detected whose abundance decreased during ageing, leaving space for the growth of subdominant species.

Further research has observed that the GM of long-lived individuals is characterised by a rearrangement in the Bacillota population, with a decline in *F. prausnitzii* and enrichment in facultative anaerobes, notably pathobionts, which correlates with an increase of the inflammatory status (Lynch and Pedersen, 2016). Similar findings have been reported from other longevity parts of the world (Kim et al., 2019; Ren et al., 2021). However, the GM of the semi-supercentenarians had greater enrichment of health-associated groups (eg. *Akkermansia*, *Bifidobacterium*, and Christensenellaceae); a key difference from the GM of centenarians.

Metagenomics has been used to examine the functions of bacteria in the GM of older people (Lynch and Pedersen, 2016). This has revealed a rearrangement in metabolic pathways related to carbohydrate and amino acid metabolism in agreement with the loss of *Eubacterium* and *Faecalibacterium* and the increase of Pseudomonadota sp. The shift from a saccharolytic to a proteolytic profile induces a marked decrease in SCFA production and availability of tryptophan and an increase in indolic metabolites, which correlate with cognitive impairment, inflammation, and cancer. The aged GM was also enriched in microorganisms capable of generating unique secondary bile acids, which could be involved in reducing the risk of infection with pathobionts (Sato et al., 2021). Interestingly, compared with younger individuals, the GM of the Italian elderly aged over 100 years had more genes for xenobiotic metabolism, particularly for chemicals deriving from the industrial manufacturing of many indoor products, such as synthetic fibres, resins, and synthetic leather (Lynch and Pedersen, 2016). This could reflect an adaptive response to increased exposure to these anthropic pollutants over a lifetime.

Looking specifically at GM characteristics that could be a marker of longevity, Christensenellaceae is worthy of further study as it is more abundant in long-lived people independent of their culture, diet, and lifestyle (Kong et al., 2016; Tuikhar et al., 2019). Research in different age groups has revealed that a greater abundance of Christensenellaceae is associated with lower body mass index, visceral adipose tissue and inflammation, more favourable lipid traits (lower total cholesterol, Apo B levels, triglycerides), and higher levels of faecal SCFAs (Waters and Ley, 2019). Hence Christensenellaceae could be a future candidate as probiotic.

Dietary modification could also encourage the acquisition of beneficial species for healthier ageing. In the NU-AGE study, Ghosh et al. (2020) recruited 1,250 healthy, pre-frail adults aged 65–79 from five European countries and randomised them to a 12-month nutritional intervention consisting of a Med diet with vitamin D supplementation versus a control diet. The GM was analysed in 612 participants before and after the intervention. Adherence to the intervention diet enriched specific GM taxa that were positively associated with cognitive function markers and negatively associated with frailty and inflammatory markers, including C-reactive protein and interleukin-17. The diet-modulated GM changes were also associated with increased SCFAs and lower production of secondary bile acids.

In summary, age group separation of the GM composition is evident, and longevity adaptation seems linked to the enrichment of health-associated GM species, including *Akkermansia*, Christensenellaceae, *Bifidobacterium*, and Odoribacteraceae, involved in the establishment of new homeostasis. These bacterial taxa could be promoted using dietary interventions to improve the “health span” of the elderly.

## Probiotics and the ageing immune system

The final presentation of the Yakult International Symposium was given by Dr Caroline Childs at the University of Southampton, UK, who examined the role of the GM in immunosenescence.

### How does the immune system age?

The thymus is responsible for manufacturing immune cells, such as T-cells, but this ability declines sharply with age after the peak thymus activity in childhood. By age 50, active thymus tissue is significantly replaced with adipose cells, resulting in lower production of naïve immune cells and a higher proportion of memory T-cells with a low functional capacity. The function of immune cells *in vitro* correlates with clinical outcomes, so it is no surprise that the coronavirus pandemic – representing a novel immune challenge – disproportionately affected older populations. Ageing is characterised by chronic, low-level inflammation (inflammageing) and a greater risk of morbidity and mortality. Older people are more likely to get infections, and their immune system responds less effectively to these and vaccinations, for example, only 30–50 per cent of elderly adults gain protection from influenza vaccinations (Demicheli et al., 2018).

T-cells fall into two categories; cytotoxic T-cells which fight infections, and helper T-cells which act like project managers. However, T-cell ageing is not automatically linked to chronological age. Some 70-year-olds may have the T-cell functionality of 30-year-olds, and vice versa (Kaczorowski et al., 2017), which correlates with the findings noted by previous speakers describing the overlap in GM composition for different age groups. Building on this point, a study of 178 older adults (Claesson et al., 2012) found that the faecal microbiota composition clustered by diet and with participants residing in care homes or the community. The care home GM was less diverse and correlated significantly with measures of frailty, co-morbidity, and inflammatory markers of inflammation. Interestingly, moving from the community to a care setting changed the diet immediately, but it took around a year for the GM to respond (O’Toole and Jeffery, 2015).

One key change in the ageing GM is the shift away from Bifidobacterium (Arboleya et al., 2016), a genus associated with immuno-modulatory properties. An *in vitro* study (You and Yaqoob, 2012) found that exposure of human mononuclear cells to probiotics from bifidobacteria and lactobacilli strains produced immunomodulatory effects, but the response was also significantly influenced by the age of the volunteer.

### How can immune ageing be measured?

Flow cytometry can measure and differentiate immune cells from human samples and determine immune age by looking at the relative proportions of naïve cells and different types of memory cells, that is, central, effector or terminally differentiated effector. Accumulation of T EMRA cells is characteristic of ageing. Other biomarkers of cell ageing include the CD28 marker on T-cells, which helps to stabilise their interaction with B cells, the source of antibody production. CD28 is progressively lost with ageing while, in contrast, the CF57 marker, which is linked to the immune response to viruses and cancer cells, appears on the T-cells and natural killer (NK) cells of older adults. This is thought to indicate cell exhaustion. A study (Tae Yu et al., 2015) in patients the morning after having a myocardial infarction revealed that the frequency of CD57 in their CD8 T-cell population positively correlated with cardiovascular mortality 6 months later. In other research, CD57 was a marker of a poor NK cell response to influenza vaccination in older subjects which could not be offset by supplementation with a synbiotic containing *B. longum* (Przemska-Kosicka et al., 2018).

Another marker of immune ageing is T-cell receptor excision circles (TRECs). These circles of DNA form when T-cells are created in the thymus and are exported to the cell cytosol. TRECs decline in concentration with each round of cell division as T-cells replicate and mature (Lang et al., 2011). Hence, one may see more TRECs in younger people and those with younger immune systems than in older or immunosenescent people (Mitchell et al., 2010). Seropositivity to viruses which disrupt immune function, such as cytomegalovirus or even SARS-Covid-19, is also a helpful marker.

Probiotics are beneficial for immune function as they lower the burden of certain infections and reduce antibiotic use (Hao et al., 2015), potentially saving health systems millions of Euros (Lenoir-Wijnkoop et al., 2015). However, the data have a high level of heterogeneity, lowering the overall quality of evidence. A review of the impact of probiotics, prebiotics, and synbiotics on immune response in older adults found evidence of improved vaccine responsiveness, NK cell activity and phagocytosis, and a reduced incidence of infections (Childs and Calder, 2017). However, only two studies used specific markers of immunosenescence, reporting increases in naïve T cells and TRECs after probiotics, and a third of the studies were not RCTs.

A SRMA of six eligible trials (Gui et al., 2020) found that probiotic use ranging from 3 to 12 weeks significantly increased NK cell activity in healthy older adults but concluded that the overall results were insufficiently convincing given the small sample sizes and very large heterogeneity. A systematic review (Chenhuichen et al., 2022) of nine RCTs and one secondary analysis assessed a broader range of parameters relating to immunity, metabolic health, GM, and cognitive function, finding overall benefits for probiotics and prebiotics, although the risk of bias in studies was considered high. Further studies should take account of immunological age at baseline to reduce heterogeneity and utilise markers of immune cell ageing and function.

## Conclusions

**Figure 1:**
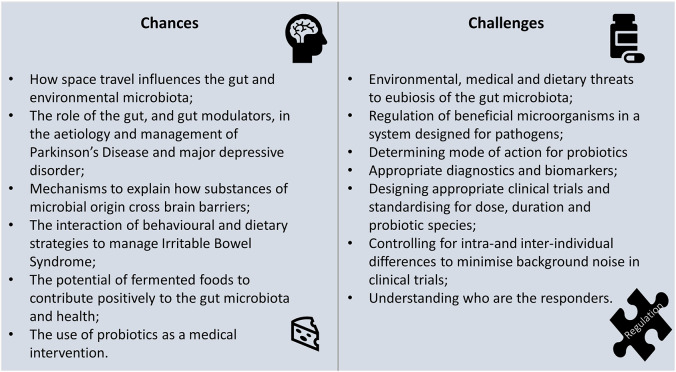
Summary of the overarching themes of the Symposium.

The evidence for the role of the GM in acute and chronic human health is now substantial, with indications that the influence of our microorganisms goes well beyond the gut to include the immune system, metabolism, and brain. While ageing and genetics impact on the composition and diversity of the GM, it is nevertheless clear that modifiable factors, such as diet, antibiotic use, exercise, and exposure to outdoor-type microbes, may be more important for achieving microbiota eubiosis. This provides people with the chance to adopt more gut-friendly lifestyles. Still, it also raises several challenges including gathering the proper evidence to ascertain which microbiota interventions are right for which population groups, understanding the mechanisms involved, developing effective probiotic and prebiotic products, and ensuring that these are appropriately regulated. As outlined in this fascinating symposium and summarised in Figure 1, there is now a tantalising opportunity to find ways to live in harmony with our GM, which could offer widespread human health benefits.

## Abbreviations

AMR: antimicrobial resistance
BBB: blood–brain barrier
BDNF: brain-derived neurotrophic factor
CSF: cerebral spinal fluid
EFSA: European Food Safety Authority
FF: fermented foods
FMT: faecal microbiota transplant
GM: gut microbiota
GRAS: generally recognised as safe
HPA: hypothalamus–pituitary–adrenal
IBS: irritable bowel syndrome
ISS: International Space Station
LAB: lactic acid bacteria
LcS: *Lacticaseibacillus paracasei* strain Shirota
LPS: lipopolysaccharide
MDD: major depressive disorder
Med: Mediterranean
NK: natural killer
OMV: outer membrane vesicles
PD: Parkinson’s disease
QPS: the qualified presumption of safety
RA: risk assessment
RCT: randomised controlled trial
RTI: respiratory tract infection
SCFA: short-chain fatty acid
SRMA: systematic review and meta-analysis
TLR: toll-like receptor
TREC: T-cell receptor excision circles
T1D: type 1 diabetes
